# Supplementary material for: A high-resolution mRNA expression time course of embryonic development in zebrafish
Source: eLife. 2017 Nov 16;6:e30860. doi: 10.7554/eLife.30860 (PMC5690287; doi:10.7554/eLife.30860)
Supplement: Supplementary file 6. [file elife-30860-supp6.zip › biolayout-clusters-files/Cluster178-genes.html]

Cluster178


# Cluster178: Genes

| Ensembl ID | Gene Name | Chr | Start | End | Biotype |
| --- | --- | --- | --- | --- | --- |
| ENSDARG00000003635 | atp6v1e1a | 5 | 37996477 | 38004317 | protein\_coding |
| ENSDARG00000014572 | chpt1 | 4 | 17718032 | 17736032 | protein\_coding |
| ENSDARG00000102981 | cyp2k16 | 3 | 12610950 | 12629309 | protein\_coding |
| ENSDARG00000019541 | gpt2l | 2 | 3701938 | 3741821 | protein\_coding |
| ENSDARG00000060153 | h6pd | 6 | 45917099 | 45927583 | protein\_coding |
| ENSDARG00000075178 | lpcat3 | 16 | 12283535 | 12345460 | protein\_coding |
| ENSDARG00000063370 | sgk2a | 23 | 4385724 | 4409136 | protein\_coding |
| ENSDARG00000059993 | trpm4a | 3 | 32360753 | 32387233 | protein\_coding |
